# A role for community-level socioeconomic indicators in targeting tuberculosis screening interventions

**DOI:** 10.1038/s41598-022-04834-7

**Published:** 2022-01-17

**Authors:** Meredith B. Brooks, Helen E. Jenkins, Daniela Puma, Christine Tzelios, Ana Karina Millones, Judith Jimenez, Jerome T. Galea, Leonid Lecca, Mercedes C. Becerra, Salmaan Keshavjee, Courtney M. Yuen

**Affiliations:** 1 Department of Global Health and Social Medicine, Harvard Medical School, 641 Huntington Avenue, Boston, MA 02115 USA; 2 Harvard Medical School Center for Global Health Delivery, Boston, MA USA; 3 Department of Biostatistics, Boston University School of Public Health, Boston, MA USA; 4 Socios En Salud Sucursal Peru, Lima, Peru; 5 School of Social Work, University of South Florida, Tampa, FL USA; 6 College of Public Health, University of South Florida, Tampa, FL USA; 7 Division of Global Health Equity, Brigham and Women’s Hospital, Boston, MA USA

**Keywords:** Tuberculosis, Health services, Public health

## Abstract

Tuberculosis screening programs commonly target areas with high case notification rates. However, this may exacerbate disparities by excluding areas that already face barriers to accessing diagnostic services. We compared historic case notification rates, demographic, and socioeconomic indicators as predictors of neighborhood-level tuberculosis screening yield during a mobile screening program in 74 neighborhoods in Lima, Peru. We used logistic regression and Classification and Regression Tree (CART) analysis to identify predictors of screening yield. During February 7, 2019–February 6, 2020, the program screened 29,619 people and diagnosed 147 tuberculosis cases. Historic case notification rate was not associated with screening yield in any analysis. In regression analysis, screening yield decreased as the percent of vehicle ownership increased (odds ratio [OR]: 0.76 per 10% increase in vehicle ownership; 95% confidence interval [CI]: 0.58–0.99). CART analysis identified the percent of blender ownership (≤ 83.1% vs > 83.1%; OR: 1.7; 95% CI: 1.2–2.6) and the percent of TB patients with a prior tuberculosis episode (> 10.6% vs ≤ 10.6%; OR: 3.6; 95% CI: 1.0–12.7) as optimal predictors of screening yield. Overall, socioeconomic indicators were better predictors of tuberculosis screening yield than historic case notification rates. Considering community-level socioeconomic characteristics could help identify high-yield locations for screening interventions.

## Introduction

Despite being curable and treatable, an estimated 10 million people develop tuberculosis (TB) annually^1^. Of these 10 million, about 3 million people are missed by the health systems, meaning that a substantial proportion of individuals sick with TB are not diagnosed or given potentially life-saving treatment^1^. Individuals with undetected TB can contribute to further transmission, leading to excess disease and deaths. For this reason, active case finding—in which individuals at increased risk of disease are actively sought out and screened for disease, leading to more diagnoses and faster initiation of appropriate treatment—is a fundamental component of the strategy for TB elimination^2–4^.

Without active case-finding, many individuals with TB disease may experience missed or delayed diagnoses because they do not perceive symptoms and therefore do not seek care, because they face barriers accessing health facilities, or because health facilities rely on sputum smear microscopy, which has low sensitivity^5^. Bringing screening services into communities is a proven strategy for closing the case detection gap^6,7^ by reaching different populations than are seen at public health facilities and, over time, reducing community TB burden^8^. However, given the geographic heterogeneity of TB epidemics^9^, questions remain about the best way to target community active case-finding efforts in order to maximize the yield of TB detected and the overall impact of the intervention^10,11^. A common strategy used to target case-finding interventions is to identify areas that are known to have high TB burdens based on routine case notification data. However, while a high case notification rate may be a good indicator of high TB burden^12^, the converse may not be true. For example, an area may have low case notification rates because of a low TB burden or because the population faces economic and other social barriers to accessing care^13–15^. Thus, it is possible that people who might most benefit from community-based screening interventions may live in areas that would not be prioritized if interventions were only targeted to places with high case notification rates^16^. Additionally, because individuals who attend community-based screening programs may be different from those who seek care at local public health facilities, historic case notification rates from the health facilities might not useful for targeting community-based screening programs.

We aimed to assess the utility of using historic case notification rates to target TB active case-finding activities and determine whether other neighborhood characteristics might be more useful. Using data from a community-based screening program in Lima, Peru, we evaluated whether the neighborhoods where the greatest percentages of screened individuals were diagnosed with TB were the same neighborhoods with the highest case notification rates in previous years. In addition, we explored whether neighborhood-level demographic or socioeconomic indicators predicted the yield of TB diagnoses among screened individuals better than historic case notification rates.

## Methods

### Study design

We conducted an exploratory analysis—with an aim of informing future work—to assess whether historic case notification rates and other neighborhood characteristics could predict how many of the individuals screened by a community-based screening program would be diagnosed with TB. Our target population was all individuals who attended the community-based screening program, regardless of where they were screened or what motivated them to be screened. Our geographic unit of analysis was residential neighborhoods, a smaller unit than health facility catchment areas; this smaller unit is more relevant to the planning of community-based screening programs, which are most likely to reach people in the immediate vicinity.

### Study population

Peru is a middle-income country with an estimated TB incidence of 119 per 100,000 population^13^. Our study focuses on residents of the contiguous catchment areas of eight primary-level public health facilities in the Carabayllo district, Lima, Peru; this area had a population of about 212,000 in the 2017 census^17^. We excluded the catchment areas of four other health facilities in the less urbanized periphery of Carabayllo because key predictor data were unavailable. While TB screening and treatment are free in Peru, people nevertheless face both direct and indirect costs for seeking care, which present barriers to timely diagnosis^18^.

### TB screening program

Starting in February 2019, a community-based TB screening program was implemented in three contiguous districts of north Lima, including Carabayllo^19^. The screening program involved mobile screening units offering free chest radiography, regardless of the presence of symptoms. If the chest radiograph was abnormal, individuals underwent a physical examination and were asked to provide a sputum sample for rapid testing with GeneXpert MTB/RIF (Cepheid, Sunnyvale, CA). Individuals were diagnosed based on either a positive GeneXpert MTB/RIF result or by a physician based on clinical and radiologic evidence. All individuals diagnosed with TB were referred for treatment to their local health facility.

Mobile units were open to the public and stationed in high-traffic areas such as parks, markets, transport terminals, and outside health facilities (which tend to be in centrally located areas). To promote awareness of the screening program, a structured community engagement strategy was implemented prior to the arrival of the mobile screening unit in each community, including the incorporation of popular opinion leaders and a multimedia campaign of videos, audio vignettes, flyers, posters, community murals and jingles^20^. Prior to launch, the implementation team consulted with community leaders to define boundaries corresponding to local definitions of Carabayllo neighborhoods, which were then mapped. Seventy-four neighborhoods ranged from 0.04 to 4.36 km^2^ in area. All people attending the screening program were asked to indicate what neighborhood they lived in, aided by the maps and staff familiar with the area.

### Data sources

Outcome data were obtained from the TB screening program for people screened February 7, 2019–February 6, 2020. We restricted analysis to residents of the geographic area of interest, including those who were screened at any mobile unit site. Neighborhood-level predictor data were obtained from the 2017 census and TB treatment registers. Details of data sources and processing can be found in the Supplementary Material.

### Statistical analysis

Our outcome of interest was neighborhood “screening yield,” defined as the proportion of screened neighborhood residents who were diagnosed with TB. We used two different analytic approaches that have complementary strengths and limitations in handling the continuous neighborhood-level predictor data: binomial logistic regression and Classification and Regression Tree (CART) analysis. Binomial logistic regression is an established approach with well-characterized methods for testing model assumptions to assure validity of the resulting model. However, ascertaining relationships between the outcome and continuous predictor variables is difficult unless meaningful thresholds for categorizing predictors are determined a priori. In contrast, CART is a nonparametric method that uses recursive partitioning to search through all potential predictors and cutoff values to categorize predictors, identifying the most important predictors and their optimum predictive thresholds. However, in CART analysis there are no established methods to estimate certainty that are analogous to confidence intervals, and CART may identify predictor thresholds that are not programmatically useful.

#### Approach 1: logistic regression analysis

We used binomial logistic regression to assess univariable associations between neighborhood-level characteristics and the odds that a person will be diagnosed with TB given that they live in a neighborhood with certain characteristics. The outcome of interest was the screening yield. Neighborhood-level predictors were assessed as continuous values since we had no way to determine a priori meaningful categorizations for these demographic and socioeconomic predictors. We ran model diagnostics, including Pearson, deviance, standardized, and likelihood residuals, Cook’s distance (D), and DFBETA, for key variables and further explored any observations that were identified to be highly influential based on the Cook’s D. For any neighborhood identified as a potential influential outlier, we conducted sensitivity analyses with the neighborhood included and excluded to assess whether its inclusion in the analysis created or strengthened associations. If it did, we removed the neighborhood from the primary analyses and presented sensitivity analyses demonstrating the impact of removing these outlier neighborhoods. We also conducted a sensitivity analysis with the outcome of bacteriologically confirmed TB. Details of sensitivity analyses can be found in Supplementary Material. Additionally, we ran spatial dependence diagnostics using the Lagrange Multiplier lag and error tests to assess whether the models should include a spatial autocorrelation term. Logistic regression analyses were performed with SAS V9.4 (SAS Institute, Cary, North Carolina, USA).

#### Approach 2: classification and regression tree analysis

We conducted two neighborhood-level CART analyses with screening yield as the outcome. Approach 2a treated screening yield as a continuous outcome, allowing the CART process to define the final outcome categories based on where it split the outcome variable. Approach 2b treated screening yield as a categorical outcome, defining the top 15 neighborhoods with the highest screening yield as “high yield” and the rest as “low yield,” and then allowing the CART process to predict which yield category each neighborhood would fall into.

The models were weighted by the number of residents screened from each neighborhood. We ranked and selected the primary node and assessed the relevance of each variable in the final model. Measures of predictive importance—determined by computing the improvement measure attributable to each variable in its role as a surrogate to the primary split—were assigned to each potential predictor, entailing both marginal and interaction effects involving this variable. The data sets were split into increasingly homogenous sub-groups, using least squares method to split nodes and add smaller daughter nodes to the tree. Maximal trees were generated and pruned based on relative misclassification costs, complexity, and parsimony. Ten-fold cross-validation was performed, in which the data set was randomly split into learning and test sets. CART analysis was then applied to determine model performance and predictive accuracy in these test sets, removing the need for a validation data set.

For the final derived trees from Approaches 2a and 2b, we assessed the utility of the predictors and thresholds identified by the analyses by converting these node values into categorical predictors, which we then used in a binomial logistic regression analysis. This produced odds ratios (OR) and 95% confidence intervals (CIs) for ease of interpretation for those more familiar with association statistics and effect sizes. Sensitivity analyses were performed to assess the impact of any outlier neighborhoods, the inclusion of mathematically related variables, and restriction to bacteriologically confirmed cases (Supplementary Material). CART analysis was run using Salford Systems Data Mining and Predictive Analytics Software version 8.0 (Salford Systems, San Diego, California, USA).

### Ethics approval

This study was conducted in accordance with the U.S. Health and Human Services regulations for the protection of human subjects (HHS 45CFR 46). Informed consent was not required, as the Mass General Brigham Institutional Review Board determined that the study constituted exempt human subjects research (protocol 2019P002416).

## Results

**Figure 1:**
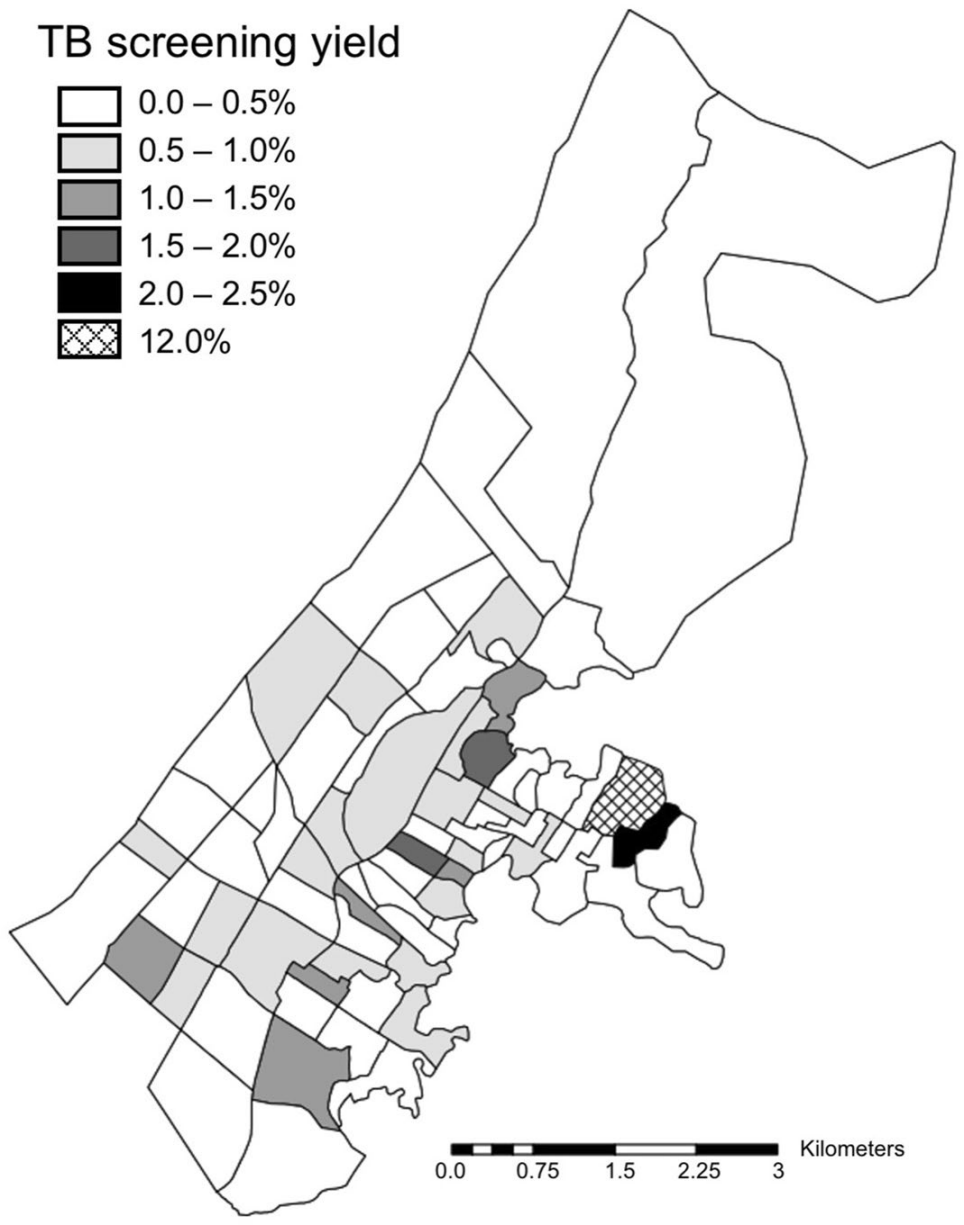
Tuberculosis screening yield by neighborhood. Map was created by MBB using ArcMap Desktop version 10.8 (Environmental Systems Research Institute, Redlands, California, USA; https://www.esri.com/en-us/arcgis/products/arcgis-desktop/).

During the analytic period, the mobile screening units screened 29,619 residents from the 74 neighborhoods (14.0% of the population) and diagnosed 147 TB cases, of which 125 (85.0%) were bacteriologically confirmed. The median TB screening yield from the screening program per neighborhood was 0.4% (interquartile range [IQR]: 0.0–0.8%; range: 0.0–2.3%, plus a single neighborhood with a much higher yield of 12.0%) (Fig. 1). The summary statistics of neighborhood characteristics are reported in Table 1.

**Table 1:** Neighborhood epidemiologic, demographic, and socioeconomic characteristics (*n* = 74 neighborhoods).

	Median	Interquartile range	Range
**Tuberculosis epidemiology**			
Historic case notification rates (annual cases per 100,000 population)			
Total	124	59–186	0–797
Male	157	75–238	0–789
Female	83	43–138	0–805
< 15 years	23	0–43	0–149
15–44 years	159	85–267	0–767
> 44 years	81	35–163	0–2191
Characteristics of historic tuberculosis patients (percent with characteristic)			
Female	38	29–47	0–100
< 15 years	6	0–11	0–20
15–44 years	69	60–78	23–100
> 44 years	20	11–26	0–55
Prior tuberculosis episode	0	0–9	0–100
**Demographics of residents**			
Population breakdown (percent of population in demographic group)			
Female	51	50–51	47–54
< 15 years	27	24–29	20–38
15–44 years	50	48–51	43–58
> 44 years	23	20–27	13–36
Neighborhood population density (residents per km^2^)			
Population density	10,000	5273–14,767	1164–19,632
** Socioeconomic indicators**			
Infrastructure (percent of occupied residential buildings with each characteristic)			
Municipal water supply	89	77–94	10–98
Informal or non-permanent structure	1	0–1	0–15
Crowding			
Individuals per residence	4.1	3.8–4.5	3.2–5.6
Households per residence	1.0	1.0–1.1	0.9–1.2
Education and occupation (percent of population with characteristic)			
Completed only primary education	30	27–32	19–52
Completed only secondary education	65	62–69	46–78
Any post-secondary education	21	16–28	10–45
Worked for pay in the past week	40	38–42	31–45
Product ownership (percent of households owning each item)			
Blender	78	72–83	60–91
Cable	56	49–64	26–79
Cellphone	91	90–94	82–98
Computer	36	27–45	13–69
Internet access	29	21–39	6–65
Iron	66	57–73	41–88
Landline	22	10–35	3–61
Microwave	27	21–35	9–60
Refrigerator	73	67–79	48–90
Sound system	49	47–54	34–67
Stove	98	97–98	90–100
Television	93	89–94	80–97
Vehicle	17	14–26	3–39
Washing machine	44	36–53	18–74

### Approach 1: logistic regression results

During assessment of model diagnostics, we identified three potentially influential observations by Cook’s D. We ran sensitivity analyses, removing each of the potentially influential observations one by one, and found that removal of one of the three neighborhoods substantially reduced the strength of several of the observed associations. This observation was a neighborhood with a 12% TB screening yield; 3 individuals were diagnosed with TB disease out of only 25 people screened. Due to this observation’s large impact on the results of the regression model, we excluded it from our primary analyses to avoid associations driven by outlier values. Thus, all primary analyses include 73 neighborhoods, and sensitivity analyses were run with all 74 neighborhoods (Supplementary Material). Additionally, no spatial dependence was observed through the Lagrange Multiplier lag and error tests, so a spatial autocorrelation term was not applied to the models.

The primary logistic regression analysis identified that the percent of households in the neighborhood that own a vehicle was strongly inversely associated with a TB diagnosis (OR: 0.76 per 10% increase in vehicle ownership; 95% CI: 0.58–0.99; *P* = 0.044) (Table 2). No other characteristics showed strong associations. The association with vehicle ownership was similar in the sensitivity analyses including the outlier neighborhood (OR: 0.70; 95% CI: 0.54–0.92; *P* = 0.011) and restricting to bacteriologically confirmed cases (OR: 0.72; 95% CI: 0.54–0.97; *P* = 0.033) (Supplementary Material).

**Table 2:** Associations between neighborhood characteristics and tuberculosis screening yield based on logistic regression (*n* = 73 neighborhoods).

Neighborhood characteristics	Median (interquartile range)	Odds ratio^a^	95% confidence interval	*P* value
**Tuberculosis epidemiology**				
Historic case notification rates (annual cases per 100,000 population)				
Total	124 (65–186)	1.04	0.83–1.30	0.753
Male	158 (83–238)	1.08	0.90–1.30	0.396
Female	87 (48–138)	0.95	0.73–1.23	0.686
< 15 years	24 (0–43)	0.83	0.49–1.39	0.473
15–44 years	160 (88–267)	1.06	0.89–1.25	0.513
> 44 years	82 (39–163)	1.00	0.88–1.14	0.958
Characteristics of historic tuberculosis patients (percent with characteristic)				
Female	38 (29–47)	0.94	0.82–1.07	0.329
< 15 years	6 (0–11)	0.81	0.57–1.13	0.218
15–44 years	69 (60–78)	1.06	0.93–1.22	0.382
> 44 years	20 (11–26)	1.01	0.87–1.18	0.887
Prior tuberculosis episode	0 (0–9)	1.04	0.94–1.16	0.395
**Demographics of residents**				
Population breakdown (percent of population in demographic group)				
Female	51 (50–51)	0.42	0.06–2.91	0.383
< 15 years	27 (24–29)	1.05	0.66–1.67	0.838
15–44 years	50 (48–51)	0.83	0.39–1.75	0.623
> 44 years	24 (20–27)	1.01	0.74–1.38	0.945
Neighborhood population density (residents per km^2^)				
Population density	10,006 (5607–13,767)	1.02	0.99–1.05	0.282
**Socioeconomic indicators**				
Infrastructure (percent of occupied residential buildings with each characteristic)				
Municipal water supply	89 (78–94)	1.06	0.96–1.17	0.270
Informal or non-permanent structure	1 (0–1)	0.85	0.26–2.76	0.787
Crowding				
Individuals per residence	4.1 (3.9–4.5)	1.02	0.99–1.05	0.287
Households per residence	1.0 (1.0–1.1)	1.06	0.87–1.28	0.586
Education and occupation (percent of population with characteristic)				
Completed only primary education	30 (27–32)	1.15	0.82–1.62	0.422
Completed only secondary education	65 (62–69)	0.89	0.66–1.21	0.467
Any post-secondary education	21 (16–28)	0.91	0.75–1.09	0.304
Worked for pay in the past week	40 (38–42)	0.91	0.49–1.70	0.772
Product ownership (percent of households owning each item)				
Blender	78 (72–83)	0.86	0.68–1.10	0.239
Cable	56 (50–64)	0.93	0.81–1.08	0.344
Cellphone	92 (90–94)	0.72	0.36–1.43	0.350
Computer	37 (27–45)	0.93	0.82–1.05	0.250
Internet access	29 (22–39)	0.95	0.85–1.06	0.362
Iron	66 (58–73)	0.94	0.80–1.10	0.433
Landline	23 (12–35)	1.01	0.91–1.11	0.912
Microwave	28 (21–35)	0.92	0.80–1.06	0.266
Refrigerator	73 (67–79)	0.89	0.74–1.08	0.251
Sound system	50 (47–54)	0.87	0.66–1.14	0.310
Stove	98 (97–98)	0.54	0.13–2.27	0.401
Television	93 (89–94)	0.97	0.61–1.55	0.903
Vehicle	17 (14–26)	0.76	0.58–0.99	0.044
Washing machine	45 (36–53)	0.95	0.84–1.07	0.402

^a^Odds ratios for population density is represented for the change in 1000 people per km^2^; odds ratios for historic case notification rates are represented for the change in 100 cases per 100,000 population; all other odds ratios are represented per 10% unit increase in the predictor variable.

### Approach 2: classification and regression tree results

Approach 2a, which treated the outcome of TB screening yield as a continuous variable, identified the top 15 most important variables for predicting TB screening yield among the 73 neighborhoods (Table 3). Fourteen out of 22 (64%) considered socioeconomic indicators were included in the 15 most important variables list, while only one out of the 16 (6%) epidemiologic or sociodemographic indicators—historic TB case notification rate amongst those greater than 44 years old—was included.

**Table 3:** Top 15 most important variables for predicting tuberculosis screening yield (Approach 2a, CART with continuous outcome; *n* = 73 neighborhoods).

Importance ranking	Variable	Relative variable importance score
1	Percent of households that own a sound system	100.0
2	Percent of households that own a blender	69.3
3	Percent of households that own a stove	55.6
4	Percent of households that own a computer	54.2
5	Percent of households that own a refrigerator	45.6
6	Percent of households that own a television	42.3
7	Percent of households that own an iron	41.9
8	Percent of households that have internet	33.5
9	Historic tuberculosis case notification rate amongst those > 44 years old	30.4
10	Percent of households that own a washing machine	25.5
11	Percent of households that own a landline phone	24.3
12	Percent of households that have cable	22.7
13	Percent of households that own a vehicle	21.4
14	Percent of population that have completed only a primary school education	20.9
15	Percent of population that have completed only a secondary school education	19.1

**Figure 2:**
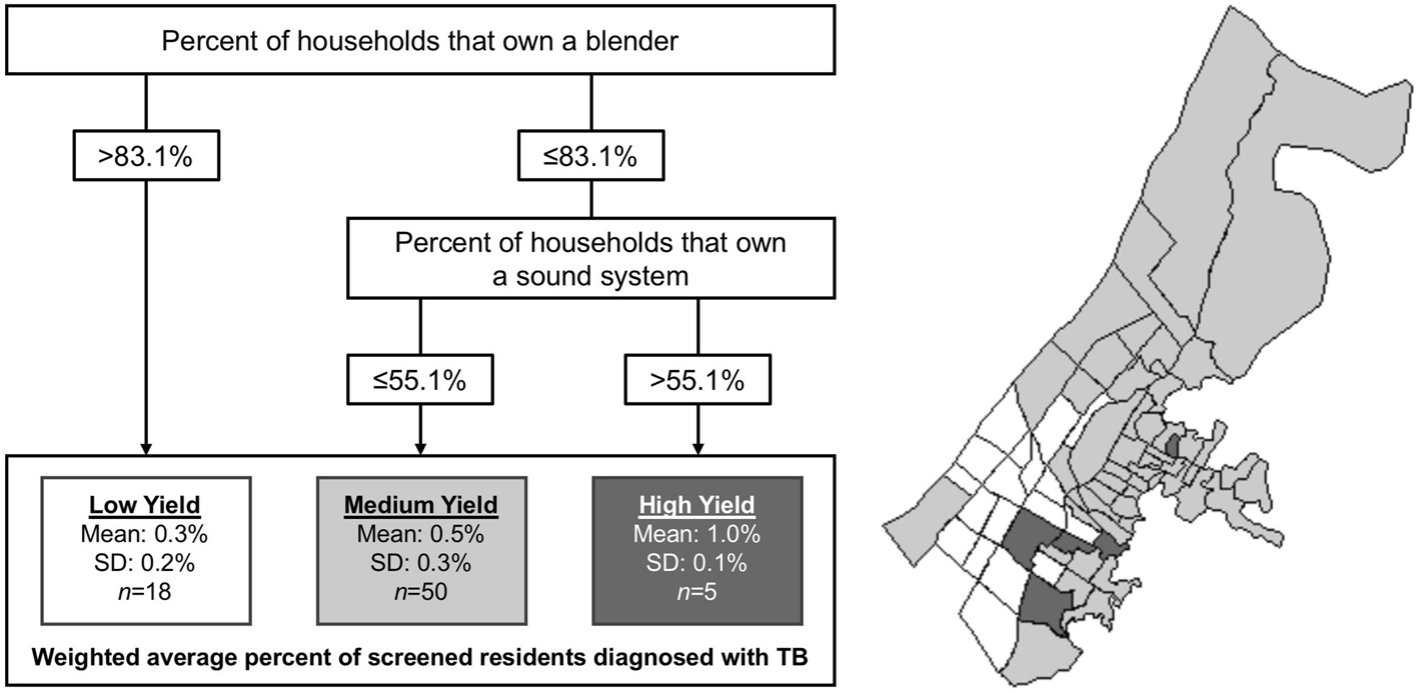
Distribution of tuberculosis screening yield according to neighborhood risk category (Approach 2a, *n* = 73 neighborhoods). Map was created by MBB using ArcMap Desktop version 10.8 (Environmental Systems Research Institute, Redlands, California, USA; https://www.esri.com/en-us/arcgis/products/arcgis-desktop/).

The primary node identified was the percent of households in a neighborhood that own a blender (Fig. 2). Neighborhoods in which ≤ 83.1% households owned a blender had a higher mean TB screening yield (mean: 0.6%, SD: 0.3%) than neighborhoods where > 83.1% of households owned a blender (mean: 0.3%, SD: 0.2%). Using this cutoff to define a categorical predictor in logistic regression found that people living in the neighborhoods with less blender ownership had 1.7 (95% CI: 1.2–2.6; *P* = 0.008) times the odds of TB compared to people living in neighborhoods with higher blender ownership.

Among neighborhoods in which ≤ 83.1% households owned a blender, those in which > 55.1% of household owned a sound system had a higher mean TB yield (mean: 1.0%, SD: 0.1%) as compared to neighborhoods where ≤ 55.1% of households owned a sound system (mean: 0.5%, SD: 0.3%). Using this cutoff to define a categorical predictor in logistic regression, we found that amongst people living in neighborhoods where ≤ 83.1 household owned a blender, those in the neighborhoods with more sound system ownership had 1.8 (95% CI: 1.1–3.0; *P* = 0.016) times the odds of TB compared to those living in neighborhoods with less sound system ownership.

Approach 2b treated the outcome of TB screening yield as a categorical variable and categorized the 15 neighborhoods with the highest screening yield as “high yield.” Across these 15 neighborhoods, the average screening yield was 1.2% (SD: 0.4%), compared to 0.2% (SD: 0.3%) in the other 58 neighborhoods. We identified the top 15 most important variables for predicting high-yield neighborhoods (Table 4). Six out of 22 (27%) considered socioeconomic indicators were included in the 15 most important variables list, while nine out of the 16 (56%) epidemiologic or sociodemographic indicators were included.

**Table 4:** Top 15 most important variables for predicting tuberculosis screening yield (Approach 2b, CART with categorical outcome; *n* = 73 neighborhoods).

Importance ranking	Variable	Relative variable importance score
1	Percent of tuberculosis patients with a prior tuberculosis episode	100.0
2	Percent of historic tuberculosis patients that were aged 15–44 years	62.3
3	Percent of households that own a vehicle	49.2
4	Proportion of the population that is female	32.5
5	Percent of population that have completed only a primary school education	31.4
6	Percent of population that have any post-secondary school education	28.2
7	Historic tuberculosis case notification rate	27.6
8	Percent of population that worked for pay in the past week	27.1
9	Percent of households that own a refrigerator	26.8
10	Percent of residences that are in informal or non-permanent structures	26.0
11	Historic tuberculosis case notification rate for individuals 15–44 years old	25.7
12	Percent of historic tuberculosis patients that were aged < 15 years	25.6
13	Historic tuberculosis case notification rate for females	24.8
14	Historic tuberculosis case notification rate for individuals > 44 years old	24.1
15	Population density (population per km^2^)	24.0

**Figure 3:**
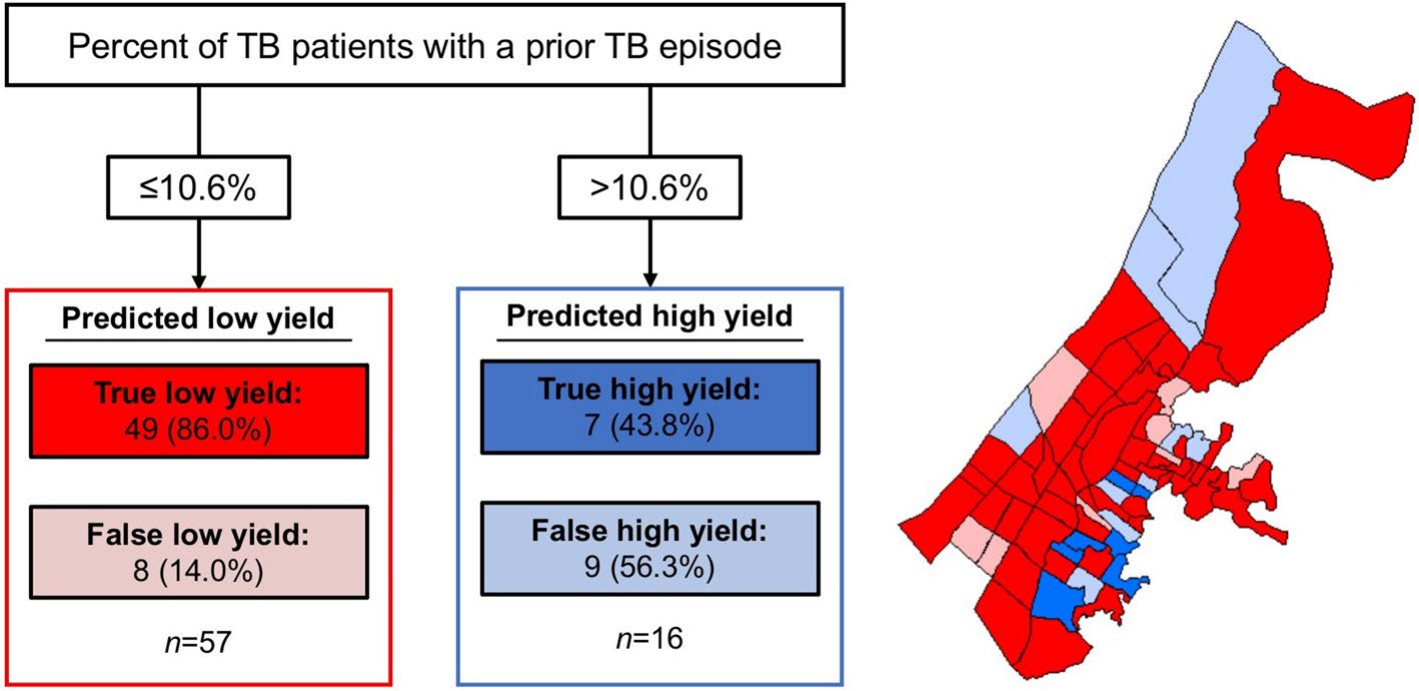
Distribution of tuberculosis screening yield according to neighborhood risk category (Approach 2b, *n* = 73 neighborhoods). Map was created by MBB using ArcMap Desktop version 10.8 (Environmental Systems Research Institute, Redlands, California, USA; https://www.esri.com/en-us/arcgis/products/arcgis-desktop/).

The primary and only node identified in the best produced tree was the percent of TB patients with a prior TB episode (Fig. 3). Greater than 10.6% of TB patients with a prior episode of TB led to the model identifying 16 neighborhoods as having a high TB screening yield, whereas 10.6% or less identified 57 neighborhoods of low TB screening yield. The positive predictive value of using the cutoff of 10.6% of historic TB patients with a history of TB as a predictor of high screening yield was 43.8% (95% CI: 19.8–70.1); the negative predictive value was 86.0% (95% CI: 77.0–95.0). We used the threshold identified by the CART analysis to define a categorical predictor variable which we subjected to logistic regression; there we found that people living in neighborhoods with greater than 10.6% of TB patients who had a prior TB episode had 3.6 (95% CI: 1.0–12.7; *P* = 0.041) times the odds of TB as compared to those living in neighborhoods with 10.6% or less of TB patients with a prior TB episode.

For both CART approaches, sensitivity analyses including the outlier neighborhood produced similar results to the primary analysis, with the same predictors identified as being most important (Supplementary Material). In the sensitivity analysis restricting to bacteriologically confirmed cases, approach 2a (continuous outcome) produced similar results as the primary analysis. Approach 2b (categorical outcome) identified the same three most important predictors as the primary analysis; the other predictors identified as important differed somewhat in the sensitivity analysis, with greater representation of socioeconomic predictors compared to the primary analysis.

## Discussion

In our study, we found that historic case notification rates were not good predictors of the yield of TB diagnoses among residents of communities served by a mobile TB screening program in Lima, Peru. In two different analytic approaches that treated screening yield as a continuous outcome, the best predictors of yield of TB diagnosis were socioeconomic indicators. This was true both when predictors were treated as continuous variables and when they were assessed for optimal partitioning via CART. Epidemiologic predictors were more useful for predicting screening yield in a CART analysis that treated the screening yield outcome as binary, although negative predictive value was far better than positive predictive value. While our analyses did not identify a single consensus set of indicators for predicting neighborhoods with high screening yields, our findings highlight the utility of considering community-level socioeconomic characteristics—rather than only historic case notification rates—when geographically targeting screening interventions.

While the association between TB and poverty is well established^21^, our findings do not simply imply that screening yields are higher in poorer neighborhoods. Although government agencies may establish income-based definitions of poverty for policy purposes, the experience of poverty is multidimensional and heterogenous^22^. Household-level poverty in Peru and other countries is best predicted by a combination of characteristics, including education, employment, housing, and ownership of certain items^23,24^. Our analysis found higher TB screening yields in neighborhoods with lower levels of vehicle and blender ownership, consistent with larger proportions of that neighborhood's residents living in poverty. However, none of our analyses identified factors related to education, employment, or housing as useful predictors. Moreover, in combination with blender ownership, we found that sound system ownership had the opposite association with TB screening yield than would be expected. Thus, the predictors identified in our analysis may be related to living in poverty, but may also reflect differences among disadvantaged communities that we are unable to explore further given the data available.

It is important to note that our outcome of screening yield is not the same as the TB prevalence in the community, as people who attend community-based screening units are not necessarily representative of the neighborhood in which they reside^25,26^. However, from a program planning perspective, screening yield is an important indicator because it can help programs launching new screening initiatives to prioritize areas where screening activities may have greater impact^27^. In addition, screening yield for a community-based program is a potential indicator of unmet need for diagnostic services, so it is meaningful even if it is not correlated with prevalence. Similarly, an association of historic case notification rates with TB screening yield may not have been observed because people attending the screening units may be fundamentally different than those who choose to present to a health facility to get diagnosed. Further, both of these groups may not be representative of the population characteristics reported in the 2017 census, which could explain the lack of association observed with many census-derived variables.

A strength of this study is the use of CART analysis, which results in easily interpretable decision trees for use in clinical practice^28,29^. CART identified previously concealed associations^30,31^, as observed when predictors included as continuous variables did not have an association with the outcome, but were associated when included as categorical variables using the thresholds identified by CART. These results can complement the results of other statistical methods; CART does not provide an analogue to a confidence interval to quantify or support the validity of the findings, but the observed thresholds can be subjected to standard hypothesis testing via regression analysis where the validity can be determined^32^. Other benefits of using CART analysis are that: it is a non-parametric method so no distributional assumptions are needed^28,29^, there is no need a priori identify hypotheses about relationships between potential predictors and the outcome, and it can overcome missing data through the use of surrogate measures^33^.

Our study was limited to the potential predictors available in the census and those routinely collected in the treatment registers. Other neighborhood-level characteristics, such as local infrastructure or accessibility to health services, may be better predictors of screening yield than those we assessed. Paper-based records and irregular address systems in many neighborhoods also limited the amount of data that could feasibly be collected on TB epidemiology indicators at the neighborhood level. Our decision to use yield as an outcome also did not take into account the varying coverage of the screening program in different neighborhoods. Additionally, our analytic population included only 147 TB cases diagnosed via a mobile screening campaign. While this corresponds to a high overall screening yield of 1 case per every 201 people screened, the number of outcome events was small for an analysis across 74 neighborhoods. This could have reduced our ability to detect associations. Due to the low absolute number of historic cases in a given neighborhood per year, we calculated a five-year average case notification rate for each neighborhood to address the potential variability over time that is not due to true changes in disease prevalence, which is in line with other studies who also aggregate cases over time to avoid issues with small case counts^34^. However, this prevented us from assessing changes in case notifications over time as a potential predictor. Finally, multiple analytic approaches were purposely used due to the complementary strengths and limitations of the methods in handling the continuous neighborhood-level predictor data. However, the use of multiple approaches led to different results, suggesting more work is needed to understand how each or both together may optimally be used to inform the optimization of community-based active case-finding for TB.

In conclusion, bringing mobile TB screening services to communities affected by poverty helps overcome barriers to accessing care. Socioeconomically disadvantaged communities may disproportionately benefit from screening interventions even if routine surveillance data does not suggest a disproportionate TB burden. Because barriers to accessing TB services can lead to underdiagnosis, limiting screening interventions to areas with known high TB burdens may exacerbate existing disparities in access to diagnostic services. Further analyses of case-finding activities should be undertaken at the neighborhood level to identify additional and better predictors of screening yield in communities.

## Supplementary Information

Supplementary Information.

## Data Availability

The data underlying this article are available in the Harvard Dataverse repository, at https://doi.org/10.7910/DVN/D8SFQY.
