# Supplementary material for: A role for community-level socioeconomic indicators in targeting tuberculosis screening interventions
Source: Sci Rep. 2022 Jan 17;12:781. doi: 10.1038/s41598-022-04834-7 (PMC8764089; doi:10.1038/s41598-022-04834-7)
Supplement: Supplementary file 1 — Supplementary Information. [file 41598_2022_4834_MOESM1_ESM.docx]

**Supplementary material for Brooks et al. “A role for community-level socioeconomic indicators in targeting tuberculosis screening interventions”**

**1. Data sources and methods for creating neighborhood-level predictors**

***Screening program data***

We used data collected by a community-based active case-finding intervention that screened attendees during February 7, 2019–February 6, 2020. To calculate the outcomes of interest, we used data on attendees’ neighborhood of residence and whether or not they were diagnosed with tuberculosis (TB) disease via the program. We used the physical maps created for the screening program to manually create neighborhood shapefiles for this analysis using ArcGIS software. The study area for this analysis comprised 74 contiguous neighborhoods.

***Census data***

We obtained the 2017 census data for the Carabayllo district of Lima, Peru, from the National Institute of Statistics and Information in Peru. The population count for the 2017 Peruvian census enumerated all people found in Lima, Peru on 22 October, 2017. There was a national stay-at-home order for urban populations on the census day to ensure people were appropriately counted. Special enumeration procedures were used for people conducting essential work on the day of the census, people living in congregate settings or who were institutionalized, and people experiencing homelessness. All people 12 years old or older were required to answer individual-level questions, while the heads of households answered questions about people less than 12 years old. A post-census quality assessment to estimate missingness identified that the census captured 94% of the population within Lima. Variables relevant to TB transmission are captured, including measures of crowing and those related to socioeconomic status. One limitation of using the census data is that it is done every 10 years and since the last census in 2007, the district lines have been redrawn. Thus, even though we are using the percent out of the total population for each indicator instead of the absolute number to ensure population growth does not impact our results, we are unable to check the stability of the indicators we are assessing over time.

Census data are aggregated by block-level census tract. Within each census tract, population numbers are available stratified by age, sex, education, and occupational status. The total number of households is also available, as well as the number of households with certain characteristics associated with socioeconomic status (e.g. access to municipal water supply, ownership of specific items).

Since the census data were aggregated by block, whereas the screening program data were aggregated by neighborhood, we needed to process the census data to match the neighborhood boundaries. Since blocks are substantially smaller than neighborhoods, but some could span more than one neighborhood, we calculated the proportion, by area, of each block-level census tract that fell into each neighborhood. We then multiplied that proportion by the census tract population and summed across each neighborhood to obtain the total population in each neighborhood. We applied the same proportions to other census data to obtain the total number of households, and the numbers of individuals or households in each neighborhood with selected demographic or socioeconomic characteristics. We then combined these numbers to calculate neighborhood-level predictors comprising the percentage of the population with certain characteristics, the percentage of households with certain characteristics, and population per residence unit.

***Historic TB case notification data***

For 2013-2017, we obtained data on the number of TB patients in the study area from the TB treatment registers of health facilities in the study area. Although routine services at the health facilities were reliant on smear microscopy for testing, patients could also be diagnosed with TB based on clinical evidence. We geocoded the addresses of each TB patient and overlaid each location onto the neighborhood-level maps. For each neighborhood, we estimated the average annual historic case notification rate during this period in two steps: first, dividing the total number of cases in each neighborhood by the 2017 estimated population calculated in that neighborhood; and second, dividing that rate by five (to give an average annual rate). We calculated age- and sex-stratified historic case notification rates for three age groups: less than 15 years, 15 to 44 years, and greater than 44 years old. For each neighborhood, we also calculated the proportion of TB patients during 2013-2017 that were in each age group, that were female, and that had reported a prior TB episode.

Shapefiles and maps were created using ArcGIS version 10.8 (Environmental Systems Research Institute, Redlands, California, USA).

**2. Sensitivity analyses of outlier neighborhoods**

***Methods***

We conducted a set of sensitivity analyses for Approaches 1, 2a, and 2b to determine the potential effect of the inclusion of any previously excluded outlier neighborhoods. Any neighborhood removed from the primary analyses due to being identified as an outlier when performing model diagnostics for the logistic regression analysis was included in these sensitivity analyses.

***Results using Approach 1: Logistic regression***

Sensitivity analyses include all 74 neighborhoods in the analytic area; this includes one neighborhood that was removed from the primary analyses because of evidence that its outlier outcome value made it unduly influential in the logistic regression. Inclusion of the outlier neighborhood did not substantially change the magnitude of the associations between individual predictors and the outcome, but tended to make the p-values smaller (Table S1).

**Table S1. Associations between neighborhood characteristics and tuberculosis screening yield based on logistic regression (*n*=74 neighborhoods)**

| **Neighborhood characteristics** | **Odds Ratio^a^** | **95% confidence interval** | ***P*-value** |
| --- | --- | --- | --- |
| **TUBERCULOSIS EPIDEMIOLOGY** | | | |
| **Historic case notification rates (annual cases per 100,000 population)** | | | |
| Total | 0.98 | 0.78 – 1.24 | 0.879 |
| Male | 1.03 | 0.86 – 1.24 | 0.729 |
| Female | 0.90 | 0.69 – 1.18 | 0.450 |
| <15 years | 0.78 | 0.46 – 1.30 | 0.338 |
| 15-44 years | 1.01 | 0.86 –1.20 | 0.866 |
| >44 years | 0.99 | 0.86 – 1.13 | 0.867 |
| **Characteristics of historic tuberculosis patients (percent with characteristic)** | | | |
| Female | 0.94 | 0.82 – 1.07 | 0.329 |
| <15 years | 0.81 | 0.57 – 1.13 | 0.218 |
| 15-44 years | 1.06 | 0.93 – 1.22 | 0.382 |
| >44 years | 1.01 | 0.87 – 1.18 | 0.887 |
| Prior tuberculosis episode | 1.04 | 0.94 – 1.16 | 0.395 |
| **DEMOGRAPHICS** | | | |
| **Population breakdown (percent of population in demographic group)** | | | |
| Female | 0.34 | 0.05 – 2.28 | 0.266 |
| <15 years | 1.22 | 0.77 – 1.92 | 0.394 |
| 15-44 years | 0.95 | 0.45 – 1.99 | 0.888 |
| >44 years | 0.92 | 0.68 – 1.26 | 0.606 |
| **Neighborhood Population density** | | | |
| Population density (per km^2^) | 1.01 | 0.98 – 1.04 | 0.508 |
| **SOCIOECONOMIC INDICATORS** | | | |
| **Infrastructure (percent of occupied residential buildings with each characteristic)** | | | |
| Municipal water supply | 1.04 | 0.94 – 1.14 | 0.439 |
| Informal or non-permanent structure | 1.07 | 0.37 – 3.08 | 0.895 |
| **Crowding** | | | |
| Individuals per residence | 1.01 | 0.98 – 1.04 | 0.427 |
| Households per residence | 1.03 | 0.85 – 1.25 | 0.795 |
| **Education and Occupation (percent of population with characteristic)** | | | |
| Primary Education | 1.25 | 0.89 – 1.74 | 0.201 |
| Secondary Education | 0.82 | 0.61 – 1.11 | 0.198 |
| Post-secondary Education | 0.87 | 0.73 – 1.05 | 0.151 |
| Worked for pay in the past week | 0.78 | 0.43 – 1.44 | 0.429 |
| **Product ownership (percent of households owning each item)** | | | |
| Blender | 0.81 | 0.64 – 1.02 | 0.073 |
| Cable | 0.91 | 0.79 – 1.05 | 0.196 |
| Cellphone | 0.71 | 0.36 – 1.39 | 0.317 |
| Computer | 0.90 | 0.79 – 1.02 | 0.099 |
| Internet access | 0.92 | 0.83 – 1.03 | 0.164 |
| Iron | 0.90 | 0.77 – 1.05 | 0.186 |
| Landline | 0.99 | 0.89 – 1.09 | 0.766 |
| Microwave | 0.90 | 0.78 – 1.03 | 0.125 |
| Refrigerator | 0.86 | 0.71 – 1.04 | 0.119 |
| Sound system | 0.80 | 0.61 – 1.05 | 0.111 |
| Stove | 0.43 | 0.11 – 1.72 | 0.232 |
| Television | 0.89 | 0.56 – 1.40 | 0.602 |
| Vehicle | 0.70 | 0.54 – 0.92 | 0.011 |
| Washing machine | 0.92 | 0.81 – 1.04 | 0.185 |

**^a^**Odds ratios for population density is represented for the change in 1,000 people per km^2^; odds ratios for historic case notification rates are represented for the change in 100 cases per 100,000 population; all other odds ratios are represented per 10% unit increase in the predictor variable.

***Results using Approach 2: Classification and regression tree analysis***

Approach 2a, which treated the outcome of TB screening yield as a continuous variable, identified the top 15 most important variables for predicting TB screening yield among the 74 neighborhoods (Table S2). These are the same 15 important predictors identified in the primary CART analysis, but in slightly different order. Fourteen out of 22 (64%) considered socioeconomic indicators were included in the 15 most important variables list, while only one out of the 16 (6%) epidemiologic or sociodemographic indicators was included, namely, historic TB case notification rate amongst those greater than 44 years old.

**Table S2. Top 15 most important variables for predicting TB screening yield (Approach 2a, CART with continuous outcome; *n*=74 neighborhoods)**

| **Importance**  **Ranking** | **Variable** | **Relative Variable**  **Importance Score** |
| --- | --- | --- |
| 1 | Percent of households that have a computer | 100.0 |
| 2 | Percent of households that have internet | 96.7 |
| 3 | Percent of households that have a landline phone | 95.3 |
| 4 | Percent of households that own a sound system | 15.8 |
| 5 | Percent of households that have a blender | 10.9 |
| 6 | Percent of households that own a stove | 8.8 |
| 7 | Percent of households that own a refrigerator | 7.2 |
| 8 | Percent of households that own a television | 6.7 |
| 9 | Percent of households that own an iron | 6.6 |
| 10 | Historic TB case notification rate amongst those >44 years old | 4.8 |
| 11 | Percent of households that own a washing machine | 4.0 |
| 12 | Percent of households that have cable | 3.6 |
| 13 | Percent of households that own a vehicle | 3.4 |
| 14 | Percent of population that have a primary school education | 3.3 |
| 15 | Percent of population that have a secondary school education | 3.0 |

The primary node identified was the percent of households in a neighborhood that own a computer (Figure S1). The identified threshold of 14.3% separated out the one neighborhood that had an outlier value of TB screening yield (12.0%), which was removed from the primary analysis, from the remaining 73 neighborhoods. The remaining nodes and thresholds were identical to that produced in the primary CART analysis.

**Figure S1. Distribution of tuberculosis screening yield according to neighborhood risk category (Approach 2a, CART with continuous outcome; *n*=73 neighborhoods)**


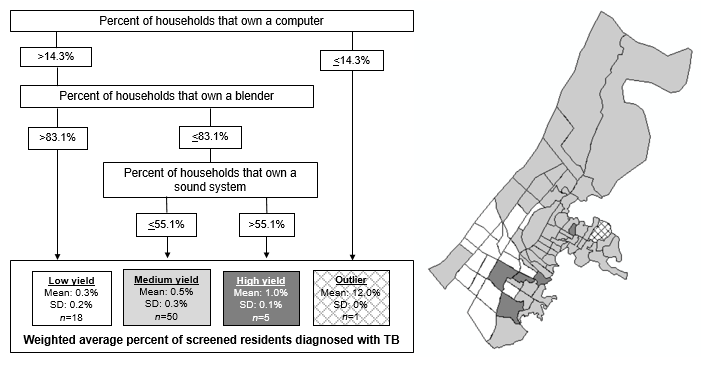


**Figure S1 Legend.** Map was created by MBB using ArcMap Desktop version 10.8 (Environmental Systems Research Institute, Redlands, California, USA; <https://www.esri.com/en-us/arcgis/products/arcgis-desktop/>).

Approach 2b treated the outcome of TB screening yield as a categorical variable and categorized the 15 neighborhoods with the highest screening yield as “high yield.” Across these 15 neighborhoods, the average screening yield was 1.9% (SD: 2.8%), compared to 0.3% (SD: 0.3%) in the other 59 neighborhoods. We identified the top 15 most important variables for predicting high-yield neighborhoods (Table S3). These are the same 15 most important predictors identified in the primary analysis, but in a slightly different order. Six out of 22 (27%) considered socioeconomic indicators were included in the 15 most important variables list, while nine out of the 16 (56%) epidemiologic or sociodemographic indicators were included.

**Table S3. Top 15 most important variables for predicting TB screening yield (Approach 2b, CART with categorical outcome; *n*=74 neighborhoods)**

| **Importance**  **Ranking** | **Variable** | **Relative Variable**  **Importance Score** |
| --- | --- | --- |
| 1 | Percent of TB patients with a prior TB episode | 100.0 |
| 2 | Percent of historic TB patients that are aged 15-44 years | 62.3 |
| 3 | Percent of households that own a vehicle | 49.7 |
| 4 | Percent of population that have a primary school education | 31.9 |
| 5 | Proportion of the population that is female | 31.5 |
| 6 | Percent of population that have a post-secondary school education | 28.7 |
| 7 | Percent of population that worked for pay in the past week | 27.6 |
| 8 | Percent of households that own a refrigerator | 27.0 |
| 9 | Historic TB case notification rate | 26.5 |
| 10 | Percent of residences that are in informal or non-permanent structures | 26.3 |
| 11 | Percent of historic TB patients that are aged <15 years | 25.6 |
| 12 | Historic TB case notification rate for females | 25.5 |
| 13 | Historic TB case notification rate for individuals 15-44 years old | 24.9 |
| 14 | Population density (population per km^2^) | 23.7 |
| 15 | Historic TB case notification rate for individuals >44 years old | 23.2 |

The primary and only node identified in the best produced decision tree was the percent of TB patients with a prior TB episode, which is the same as in the primary analysis (Figure S2). Greater than 10.6% of TB patients with a prior TB episode led to the model identifying 15 neighborhoods as having a high TB screening yield, whereas 10.6% or less identified 59 neighborhoods of low TB screening yield. The positive predictive value of using the cutoff of 10.6% of TB patients with a prior TB episode as a predictor of high screening yield is 40.0% (95% CI: 15.2-64.8) and the negative predictive value is 84.8% (95% CI: 75.6-93.9). Using the identified threshold as a cutoff to define a categorical predictor in logistic regression, we found that people living in neighborhoods with 10.6% or greater of TB patients with a prior TB episode had 1.6 (95% CI: 1.1-2.3; *P*=0.015) times the odds of TB as compared to those living in neighborhoods with less than 10.6% of TB patients having a prior TB episode.

**Figure S2. TB screening yield distribution by neighborhood-level characteristics identified via Approach 2b (CART with categorical outcome; *n*=74 neighborhoods)**


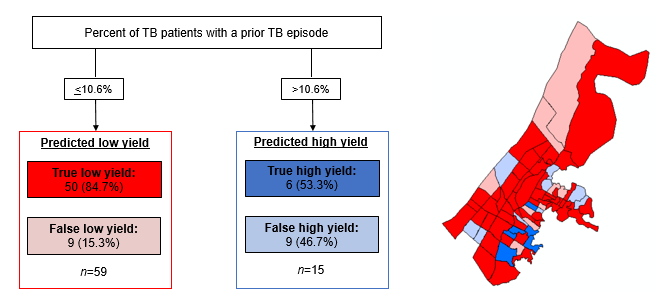


**Figure S2 Legend.** Map was created by MBB using ArcMap Desktop version 10.8 (Environmental Systems Research Institute, Redlands, California, USA; <https://www.esri.com/en-us/arcgis/products/arcgis-desktop/>).

**3. Sensitivity analyses for bacteriologically confirmed cases**

***Methods***

We conducted a set of sensitivity analyses for Approaches 1, 2a, and 2b with TB diagnoses restricted to bacteriologically confirmed cases to determine if the inclusion of cases diagnosed based on clinical or radiographic criteria is likely to have affected the conclusions.

***Results using Approach 1: Logistic regression***

Restriction to bacteriologically confirmed cases yielded similar effect estimates and p-values as the primary analysis (Table S4).

**Table S4. Associations between neighborhood characteristics and bacteriologically confirmed tuberculosis screening yield based on logistic regression (*n*=73 neighborhoods)**

| **Neighborhood characteristics** | **Odds Ratio^a^** | **95% confidence interval** | ***P*-value** |
| --- | --- | --- | --- |
| **TUBERCULOSIS EPIDEMIOLOGY** | | | |
| **Historic case notification rates (annual cases per 100,000 population)** | | | |
| Total | 1.08 | 0.85 – 1.36 | 0.546 |
| Male | 1.10 | 0.90 – 1.34 | 0.342 |
| Female | 1.01 | 0.78 – 1.31 | 0.943 |
| <15 years | 0.95 | 0.55 – 1.64 | 0.850 |
| 15-44 years | 1.09 | 0.91 – 1.31 | 0.332 |
| >44 years | 1.01 | 0.89 – 1.15 | 0.853 |
| **Characteristics of historic tuberculosis patients (percent with characteristic)** | | | |
| Female | 0.99 | 0.85 – 1.14 | 0.856 |
| <15 years | 0.90 | 0.63 – 1.30 | 0.575 |
| 15-44 years | 1.05 | 0.90 – 1.22 | 0.548 |
| >44 years | 1.01 | 0.86 – 1.20 | 0.867 |
| Prior tuberculosis episode | 1.00 | 0.89 – 1.14 | 0.943 |
| **DEMOGRAPHICS** | | | |
| **Population breakdown (percent of population in demographic group)** | | | |
| Female | 0.28 | 0.04 – 2.29 | 0.237 |
| <15 years | 1.03 | 0.62 – 1.71 | 0.910 |
| 15-44 years | 0.77 | 0.35 – 1.73 | 0.532 |
| >44 years | 1.03 | 0.73 – 1.45 | 0.852 |
| **Neighborhood Population density** | | | |
| Population density (per km^2^) | 1.02 | 0.99 – 1.06 | 0.206 |
| **SOCIOECONOMIC INDICATORS** | | | |
| **Infrastructure (percent of occupied residential buildings with each characteristic)** | | | |
| Municipal water supply | 1.03 | 0.93 – 1.14 | 0.558 |
| Informal or non-permanent structure | 1.05 | 0.33 – 3.38 | 0.933 |
| **Crowding** | | | |
| Individuals per residence | 1.17 | 0.84 – 1.64 | 0.351 |
| Households per residence | 1.32 | 0.16 – 11.1 | 0.796 |
| **Education and Occupation (percent of population with characteristic)** | | | |
| Primary Education | 1.11 | 0.77 – 1.61 | 0.580 |
| Secondary Education | 0.92 | 0.66 – 1.28 | 0.617 |
| Post-secondary Education | 0.91 | 0.75 – 1.11 | 0.367 |
| Worked for pay in the past week | 0.99 | 0.50 – 1.95 | 0.972 |
| **Product ownership (percent of households owning each item)** | | | |
| Blender | 0.86 | 0.66 – 1.12 | 0.265 |
| Cable | 0.93 | 0.80 – 1.09 | 0.374 |
| Cellphone | 0.58 | 0.29 – 1.23 | 0.157 |
| Computer | 0.93 | 0.81 – 1.06 | 0.268 |
| Internet access | 0.94 | 0.83 – 1.06 | 0.300 |
| Iron | 0.93 | 0.79 – 1.11 | 0.419 |
| Landline | 0.99 | 0.91 – 1.12 | 0.840 |
| Microwave | 0.93 | 0.80 – 1.09 | 0.363 |
| Refrigerator | 0.91 | 0.74 – 1.12 | 0.353 |
| Sound system | 0.89 | 0.66– 1.19 | 0.427 |
| Stove | 0.66 | 0.14 – 3.19 | 0.607 |
| Television | 0.92 | 0.55 – 1.52 | 0.732 |
| Vehicle | 0.72 | 0.54 – 0.97 | 0.033 |
| Washing machine | 0.95 | 0.83 – 1.09 | 0.469 |

**^a^**Odds ratios for population density is represented for the change in 1,000 people per km^2^; odds ratios for historic case notification rates are represented for the change in 100 cases per 100,000 population; all other odds ratios are represented per 10% unit increase in the predictor variable.

***Results using Approach 2: Classification and regression tree analysis***

Approach 2a, which treated the outcome of bacteriologically confirmed TB screening yield as a continuous variable, identified the top 15 most important variables for predicting TB screening yield among the 73 neighborhoods (Table S5). Thirteen out of the 15 were the same predictors that were ranked most important in the primary analysis. Twelve out of 22 (55%) considered socioeconomic indicators were included in the 15 most important variables list, while only 3 out of the 16 (19%) epidemiologic or sociodemographic indicators were included.

**Table S5. Top 15 most important variables for predicting bacteriologically confirmed TB screening yield (Approach 2a, CART with continuous outcome; *n*=73 neighborhoods)**

| **Importance**  **Ranking** | **Variable** | **Relative Variable**  **Importance Score** |
| --- | --- | --- |
| 1 | Percent of households that own a sound system | 100.0 |
| 2 | Percent of households that own a computer | 82.8 |
| 3 | Percent of households that own a blender | 63.2 |
| 4 | Percent of households that own a washing machine | 63.1 |
| 5 | Percent of households that own an iron | 56.7 |
| 6 | Percent of households that own a television | 51.8 |
| 7 | Percent of households that own a refrigerator | 43.6 |
| 8 | Percent of households that have internet | 37.7 |
| 9 | Percent of households that own a stove | 32.3 |
| 10 | Percent of population that have a secondary school education | 31.8 |
| 11 | Percent of population that have a primary school education | 30.8 |
| 12 | Percent of households that own a vehicle | 27.6 |
| 13 | Historic TB case notification rate for individuals 15-44 years old | 25.6 |
| 14 | Historic TB case notification rate for individuals >44 years old | 24.6 |
| 15 | Proportion of the population that is <15 years old | 24.5 |

Approach 2b treated the outcome of bacteriologically confirmed TB screening yield as a categorical variable and categorized the 15 neighborhoods with the highest screening yield as “high yield.” Of these, 14 were the same neighborhoods with the highest screening yields identified in the primary analysis. We identified the top 15 most important variables for predicting high-yield neighborhoods (Table S6). Seven out of the 15 most important predictors were the same as in the primary analysis, including the top three in the same order. Nine out of 22 (41%) considered socioeconomic indicators were included in the 15 most important variables list, while six out of the 16 (38%) epidemiologic or sociodemographic indicators were included.

**Table S6. Top 15 most important variables for predicting bacteriologically confirmed TB screening yield (Approach 2b, CART with categorical outcome; *n*=73 neighborhoods)**

| **Importance**  **Ranking** | **Variable** | **Relative Variable**  **Importance Score** |
| --- | --- | --- |
| 1 | Percent of TB patients with a prior TB episode | 100.0 |
| 2 | Percent of historic TB patients that are aged 15-44 years | 74.0 |
| 3 | Percent of households that own a vehicle | 50.9 |
| 4 | Percent of population that have a primary school education | 47.9 |
| 5 | Historic TB case notification rate for individuals >44 years old | 46.8 |
| 6 | Proportion of the population that is >44 years old | 40.5 |
| 7 | Percent of households that own a stove | 39.7 |
| 8 | Proportion of the population that is <15 years old | 38.1 |
| 9 | Percent of households that own an iron | 35.2 |
| 10 | Historic TB case notification rate for women | 35.0 |
| 11 | Percent of households that have internet | 32.5 |
| 12 | Percent of households that own a sound system | 32.1 |
| 13 | Percent of households that own a computer | 29.4 |
| 14 | Percent of population that have a secondary school education | 28.5 |
| 15 | Percent of population that have a post-secondary school education | 28.5 |

**4. Sensitivity analyses for mathematically related predictors**

Another set of sensitivity analyses was conducted for only Approaches 2a and 2b to ensure that the inclusion of mathematically related predictors did not affect the results.  Because the percentage of the population that is <15, 15-45, and >45 years old must sum to 100%, including two of these variables in a decision tree would eliminate the information content of the third. We therefore conducted sensitivity analyses removing one of the three predictors to assess whether this relatedness affected the results. Sensitivity analyses removing one of the population age structure predictors yielded the same relative variable importance scores and decision trees in the primary CART analysis (Tables 3 and 4 and Figures 2 and 3 in the main text).
